# The abundances of LTF and SOD2 in amniotic fluid are potential biomarkers of gestational age and preterm birth

**DOI:** 10.1038/s41598-023-31486-y

**Published:** 2023-03-25

**Authors:** Te-Yao Hsu, Hsin-Hsin Cheng, Kuo-Chung Lan, Hsuan-Ning Hung, Yun-Ju Lai, Chih-Chang Tsai, Wen-Lang Fan, Sung-Chou Li

**Affiliations:** 1 Department of Obstetrics and Gynecology, Kaohsiung Chang Gung Memorial Hospital and, Chang Gung University College of Medicine, Kaohsiung, Taiwan; 2 Department of Obstetrics and Gynecology, Jen-Ai Hospital, Taizhong, Taiwan; 3 Department of Medical Research, Kaohsiung Chang Gung Memorial Hospital and Chang Gung University College of Medicine, 12th Floor, Children’s Hospital, No.123, Dapi Rd, Niaosong District, Kaohsiung, 83301 Taiwan; 4 Department of Medical Education and Research, Kaohsiung Veterans General Hospital, 4th Floor, No.386, Dazhong 1st Rd, Zuoying District, Kaohsiung, 813414 Taiwan

**Keywords:** Biomarkers, Medical research

## Abstract

Neonates who are born preterm (PT) are usually characterized by immature physiological development, and preterm birth (PTB) is the leading cause of neonatal morbidity and mortality if intensive medical care is not available to PTB neonates. Early prediction of a PTB enables medical personnel to make preparations in advance, protecting the neonate from the subsequent health risks. Therefore, many studies have worked on identifying invasive or noninvasive PT biomarkers. In this study, we collected amniocentesis-derived (at the second trimester of gestation) amniotic fluid (AF) samples. At delivery, AF samples were classified into PTB or full-term birth (FTB). We first applied protein mass spectrometry technology to globally screen AF proteins, followed by specific protein validation with ELISA. We identified four protein biomarkers of PTB, including lactotransferrin (LTF), glutathione-disulfide reductase (GSR), myeloperoxidase (MPO) and superoxide dismutase 2 (SOD2). Further analyses demonstrated that their abundances were negatively correlated with neonatal weight and gestational age. In addition, by mimicking survival rate analysis widely used in tumor biology, we found that LTF and SOD2 were prognostic factors of gestational age, with higher levels denoting shorter gestational age. Finally, using the abundances of the four protein biomarkers, we developed a prediction model of PTB with an auROC value of 0.935 (sensitivity = 0.94, specificity = 0.89, *p* value = 0.0001). This study demonstrated that the abundances of specific proteins in amniotic fluid were not only the prognostic factors of gestational age but also the predictive biomarkers of PTB. These four AF proteins enable identification of PTB early in the second trimester of gestation, facilitating medical intervention to be applied in advance.

## Introduction

Preterm birth (PTB) is the leading cause of neonatal morbidity and mortality^1^. The definition of PTB is birth before 37 weeks of gestation^2^. The incidence of PTB in developed countries ranges from 5 to 10%, whereas it is 25% in developing countries^3^. Approximately 10% of births in Taiwan were preterm in 2009^4^. There are several major pathogenic mechanisms of PTB, including stress-induced activation of the fetal hypothalamic-pituitary-adrenal axis^5^, infections^6^, decidual hemorrhage^7^ and pathologic uterine distention^8^. Metabolomics^9^, genetic and environmental factors^10^ were also discussed as pathogenesis of PTB recently. Patients with PTB will face preterm complications, including neonatal mortality and morbidity, respiratory distress syndrome, intraventricular hemorrhage and necrotizing enterocolitis^11^. These complications result in a major economic burden on countries.

In the past years, many studies have worked on identifying antenatal prediction biomarkers of PTB and these identified biomarkers belonged to protein abundance, mRNA level or physiological measurements. Hornaday et al^12^*.* made systematic review to identify maternal blood biomarkers of PTB. Although they reviewed seventy-seven primary research articles, there was no single biomarker able to clearly predict PTB. Current laboratory PTB prediction methods depend on fetal fibronectin, placental alpha-microglobulin-1 and phosphorylated insulin-like growth factor binding protein-1^13,14^. However, limited studies have demonstrated their efficacy, and there is still no accurate method to predict PTB.

Amniotic fluid can protect the fetus from mechanical trauma, prevent infection, act as nutrients and help fetal lung and musculoskeletal maturation^15,16^. Bacteria, lower glucose concentrations, higher white cell counts, higher concentrations of complement C3 and various cytokines were proven to be pathogenetic factors in women with PTB^17^. In our previous study, we conducted traditional proteomics and concluded that apolipoprotein A-IV, lumican and kininogen-1 in the amniotic fluid were potential biomarkers of PTB^18^. However, owing to the limitation of traditional proteomics method, the number of identified proteins was limited. In addition, we failed to develop a prediction model of PTB based on these potential biomarkers. To continue our research on amniotic fluid, the purpose of our article is to propose a prediction model for the amniotic fluid of PTB patients. By global screening with gel-free protein mass spectrum technology and specific validation with ELISA, we identified four preterm biomarkers and developed a high-performance prediction model of PTB.

## Methods

### Overview of enrolled participants

**Figure 1:**
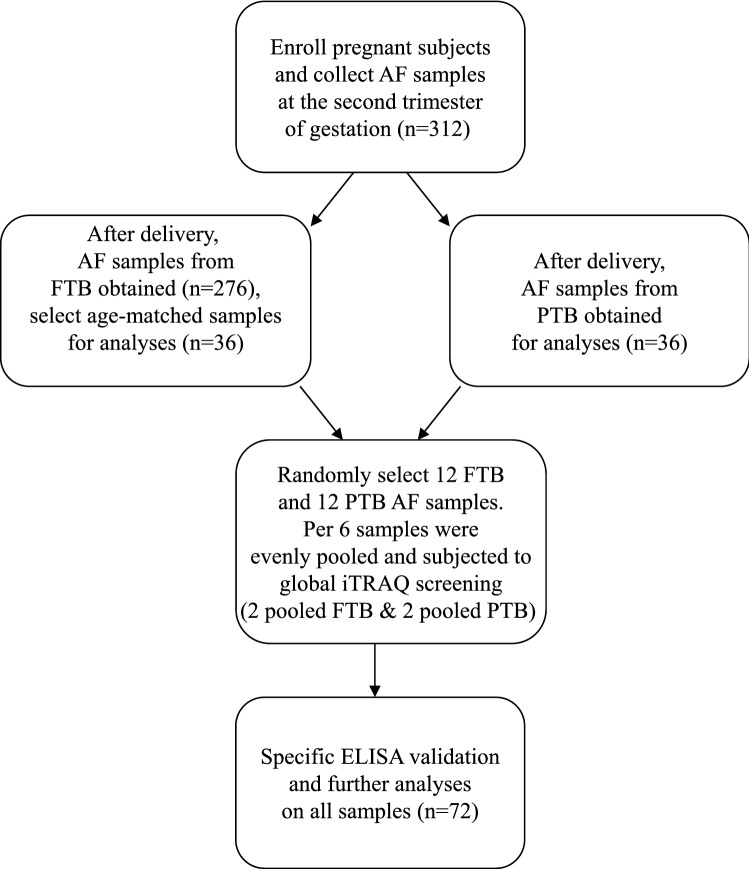
The overall flowchart of this study. We used a flowchart to illustrate the overall workflow and to direct the numbers of samples size, collected and analyzed.

This study was approved by the institutional review board of Chang Gung Memorial Hospital. All methods were performed in accordance with the relevant guidelines and regulations. All subjects or their guardians signed informed consent forms. Pregnant participants were enrolled without considering the gestational age in advance and they were further classified into FTB or PTB after delivery (Fig. 1). The exclusion criteria are fetal anormaly, multiple pregnancy and preeclampsia.

Amniocentesis is used to detect Down syndrome, chromosomal anomalies and other genetic diseases. We collected AF samples (10 ml) from each participant who underwent amniocentesis during the second trimester of gestation (week 16 ~ week 18) in our hospital. And, the gestational ages of participants were determined with an ultrasound examination and by counting the days after the last menstrual period as suggested by the American Congress of Obstetricians and Gynecologists^19^. We used centrifugation to remove the amniocytes and to enrich the cell-free supernatant. Then, the amniotic fluid samples were stored at − 80 °C until use.

### Protein identification with iTRAQ proteomics from amniotic fluid

In this study, we used isobaric tag for relative and absolute quantitation (iTRAQ) gel-free proteomics to identify and quantify the proteins in amniotic fluid samples by referring to our previous studies^20,21^. In summary, we randomly selected 12 PTB AF samples and 12 FTB AF samples, followed by measuring the concentration of total protein. Then, per six AF samples with equal amounts of total protein were evenly pooled into one tube. As a result, two pooled PTB and two pooled FTB AF samples were acquired. Then, the four pooled protein samples were prepared with the standard protocol of the iTRAQ Reagents Multiplex Kit (4352135, Sciex). Next, the labeled samples passing the QC check were analyzed with LC/Q-Exactive Orbitrap MS (Thermo), followed by raw data analysis with Proteome Discoverer v2.4 (Thermo) using the MASCOT 2.5 database (Matrix Science). The detected protein abundance profiles were further analyzed with Partek to calculate *p* values (FTB vs. PTB).

### Protein validation with ELISA

Six proteins detected with iTRAQ were further validated with ELISA in 36 FTB and 36 PTB AF samples. We operated ELISA by referring to the standard protocols of the manufacturers. The commercial ELISA kit for the six proteins are as follow: lactotransferrin (ab200015, Abcam), glutathione-disulfide reductase (OKEH01478, Aviva Systems Biology), myeloperoxidase (ab119605, Abcam) and superoxide dismutase 2 (ab178012, Abcam), insulin Like 4 (OKEH04422, Aviva Systems Biology) and cystatin 2 (OKDD00213, Aviva Systems Biology).

### Statistical analyses

In this study, most numerical and categorical data were calculated based on t-test and Chi-square test, respectively. To examine the correlation, we used Microsoft Excel program to calculate the correlation coefficients between the concentrations of four proteins and the values of three clinical manifestations, including subject age, neonatal weight and gestational age. Since PTB is defined based on gestational age at delivery, we also conducted survival rate analysis by mimicking survival time with gestational age to determine whether the protein biomarkers contribute to the prognosis of gestational age. This analysis was performed with SPSS (version 20.0, SPSS, Chicago, IL, USA) and the *p* values were calculated with the log-rank test.

To develop a prediction model of PTB, we applied Support vector machine (SVM) which is one type of machine learning algorithm and is good in dealing with binary questions, e.g. disease vs. health, treatment vs. control and so on. We inputted the ELISA-confirmed proteins into the SVM to develop a protein-based prediction model of PTB. Meanwhile, we first used a tenfold cross-validation method to reach the prediction model with two parameters determined (gamma = 64 and cost = 1)^20,22^. When one new unknown case comes in, the SVM prediction model can quickly determine whether this case is a PTB or FTB.

### Deriving the possible functions and interactions of biomarkers

In addition to facilitating PTB prediction, we were also interested in the possible functions and interactions of the four biomarkers of PTB. Therefore, we had the four biomarkers analyzed with Ingenuity Pathway Analysis (IPA, Qiagen).

### Ethics approval and consent to participate

This study was approved by the institutional review board of Chang Gung Memorial Hospital (IRB number: 99-3890B and 102-5739B). All subjects or their guardians signed informed consent forms.

## Results

### Overview of enrolled participants

We enrolled pregnant subjects to participate in this study. The overall workflow and the numbers of collected samples and analyzed samples are illustrated in Fig. 1. The clinical manifestations of 36 FT birth and 36 PT birth subjects are shown in Table 1. The maternal age, gravidity, parity and delivery method factors did not reach significant difference between the two sets. As expected, the PT birth participants had significantly lower values of gestational age (39.34 ± 0.88 vs. 32.13 ± 4.64, *p* value < 0.0001) and neonatal weight (3,355.56 ± 361.23 vs. 1,896.14 ± 814.57, *p* value < 0.0001). Among the 36 PTB subjects, 25 are preterm premature rupture of membrane (PPROM) and 11 are preterm labor (PTL) cases. And, all enrolled cases are singletons.

**Table 1:** Demographic data of participants and comparisons of clinical manifestations

	Full-term birth, n = 36	Preterm birth, n = 36	*p* value
Maternal age (year)	34.70 ± 2.25 (2.70)	35.91 ± 3.09 (4.60)	NS
Gestational age at delivery (week)	39.34 ± 0.88 (1.00)	32.13 ± 4.64 (6.86)	< 0.0001
Gravidity	2.25 ± 0.91 (1.00)	2.19 ± 1.64 (2.00)	NS
Parity	1.81 ± 0.62 (1.00)	1.53 ± 0.88 (1.00)	NS
Neonatal weight (gram)	3,355.56 ± 361.23 (565.00)	1,896.14 ± 814.57 (1245.00)	< 0.0001
Delivery method (C/S; NSD)	(9; 27)	(15; 21)	NS

We tabulated demographic data and compared the clinical manifestations of preterm and FTB participants. The first five *p* values were calculated with a t test. The *p* value of the delivery method was calculated with the chi-square test. C/S and NSD denote cesarean section and normal spontaneous delivery, respectively. NS denoted no significance. Numerical data were presented as mean ± SD with interquartile range (IQR) highlighted in bracket. The two values separated by semicon in the brackets denoted the numbers of participants with cesarean section and normal spontaneous delivery, respectively.

### iTRAQ identified proteins differentially abundant between PTB and FTB amniotic fluid samples

**Figure 2:**
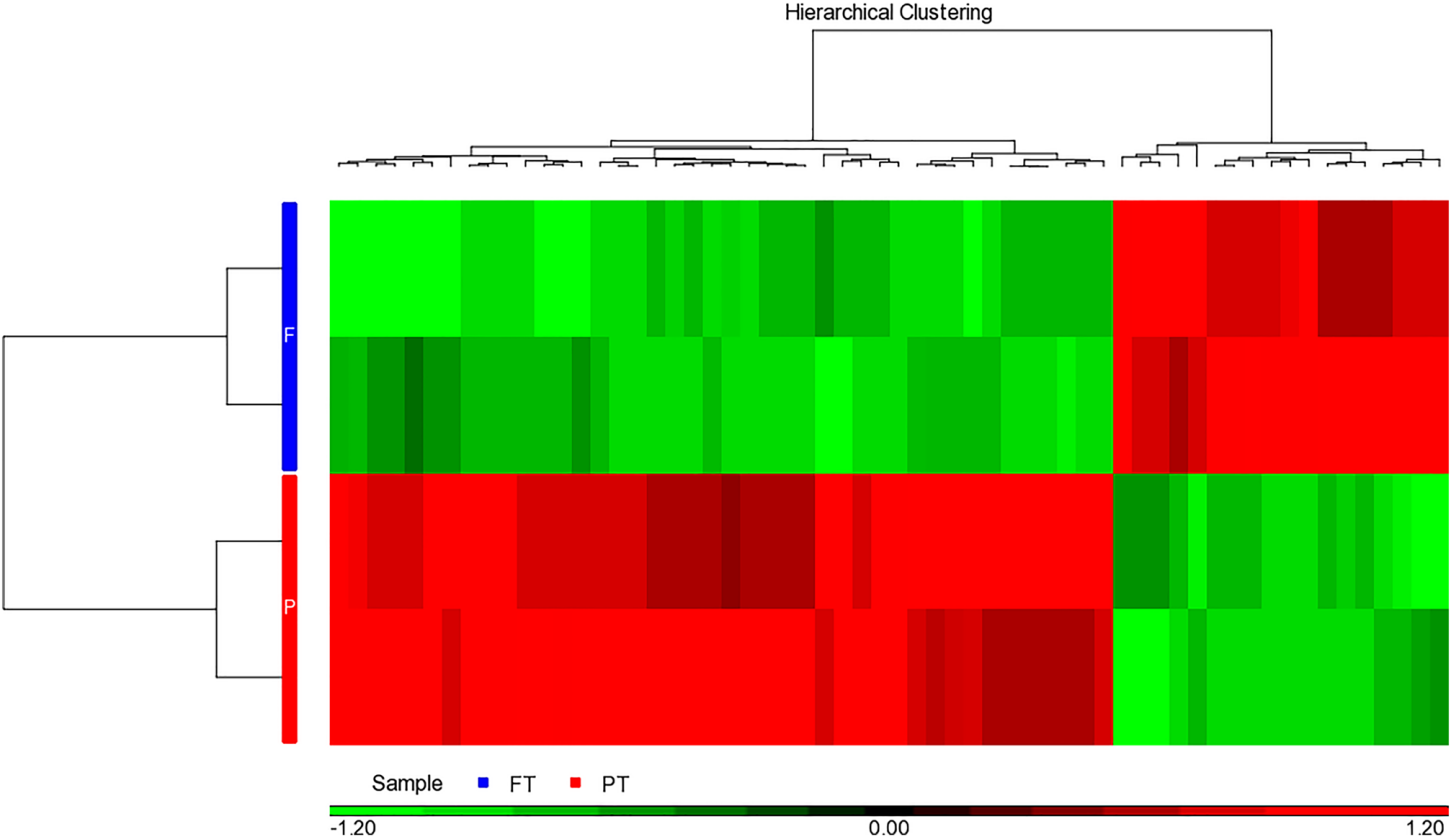
The presentation of 60 amniotic proteins differentially abundant between the preterm (PT) and full-term (FT) sets. PT and FT denoted preterm and full-term infants, respectively. With the criterion of a *p* value < 0.05 and abundance fold-change > 1.25, 60 proteins reached statistical significance. Among them, 42 remained at higher levels in preterm samples (the red pixels in preterm lanes) and 18 remained at lower levels in preterm samples (the green pixels in preterm lanes).

By global screening with iTRAQ assay, we totally identified 1,029 proteins from amniotic fluid samples with the following parameters specified: protein and peptide identification with a false determinate rate < 0.01 and the proteins identified by at least one unique peptide. Further ANOVA analysis identified 60 differentially abundant proteins when the criteria of *p* < 0.05 (t test) and variation fold-change > 1.25 were set (PTB vs. FTB). The abundance profiles of these 60 proteins are plotted in Fig. 2. As shown in Fig. 2, 70% of these proteins remained at higher levels in PTB samples and 30% of them remained at higher levels in FTB samples.

We further used Gene Ontology (GO) analysis to examine the functions of these 60 proteins. Table 2 demonstrates the top 10 most significant GO items. Most GO items were extracellular-related GO functions, which highlighted the fact that the proteins were collected from extracellular liquid biopsy, namely amniotic fluid.

**Table 2:** The gene ontology (GO) analysis results for the 60 differentially abundant proteins

Function	Type	Enrichment *p* value	GO ID
Extracellular region part	Cellular component	2.24E−27	44,421
Extracellular organelle	Cellular component	1.03E−21	43,230
Extracellular membrane-bounded organelle	Cellular component	1.03E−21	65,010
Extracellular vesicular exosome	Cellular component	1.03E−21	70,062
Membrane-bounded vesicle	Cellular component	3.10E−19	31,988
Vesicle	Cellular component	1.28E−18	31,982
Extracellular matrix	Cellular component	6.61E−09	31,012
Extracellular space	Cellular component	1.55E−07	5615
Extracellular matrix organization	Biological process	1.67E−06	30,198
Extracellular structure organization	Biological process	1.70E−06	43,062

We conducted GO analysis on the 60 differentially abundant proteins. Only the 10 most significant GO functions are shown.

### ELISA validation identified PTB biomarkers

**Figure 3:**
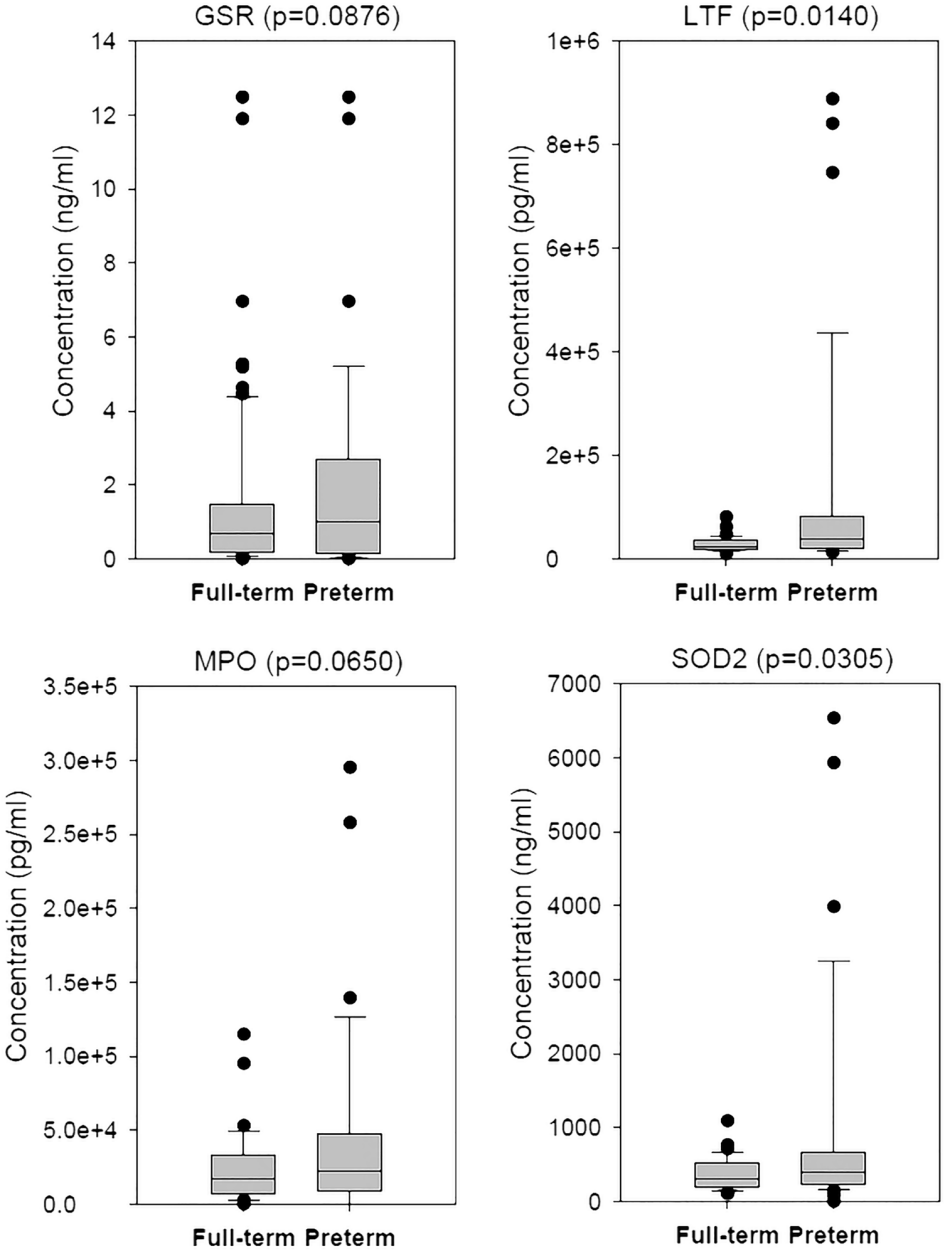
The ELISA results for specific proteins. We used ELISA to validate six candidate proteins screened by mass spectrum data and used box plots to illustrate the concentrations of four proteins.

Among these 60 proteins, we chose six for ELISA validation in 36 FTB and 36 PTB AF samples. As a result, lactotransferrin (LTF) and superoxide dismutase 2 (SOD2) were significantly differentially abundant (Fig. 3) so that they were regarded as PTB biomarkers for further assays. Although glutathione-disulfide reductase (GSR) and myeloperoxidase (MPO) did not reach statistical significance (*p* < 0.05), they were close to statistical significance. Therefore, they were also included in the further assays. Table 3 tabulates the detailed concentrations of these four proteins and all of them kept higher levels in PT AF samples.

**Table 3:** The abundances of four biomarker proteins in full-term and preterm sets.

	MPO (pg/ml)	LTF (pg/ml)	SOD2 (ng/ml)	GSR (ng/ml)
Full-term	24,562.8 ± 4,122.5	27,655.7 ± 2.335.1	372.8 ± 36.8	1.1 ± 0.2
Preterm	46,817.8 ± 1,1133.5	122,149.6 ± 37,414.6	938.4 ± 253.5	2.0 ± 0.5
P value	0.0650	0.0140	0.0305	0.0876

We determined the concentrations of MPO, LTL, SOD2 and GSR in amniotic fluid with ELISA as well as the *p* values. Data are presented as the mean ± SD. The *p* values were calculated based on t test.

### Correlations between protein biomarkers and clinical manifestations

**Figure 4:**
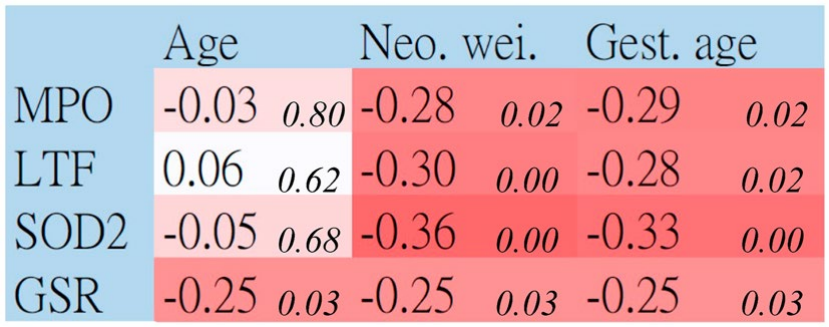
Correlations and *p* values between protein concentrations and clinical manifestations. We examined whether the concentrations of four protein biomarkers were correlated with the clinical manifestations by conducting correlation analysis on all 72 participants. Age denoted maternal age at delivery. In each column, the left digit and right digit (in italic style) denote the correlation coefficient and *p* value, respectively. The deeper red pixels denote higher correlation coefficients.

Since the abundances of the four protein biomarkers were significantly or almost significantly varied between the two sets, we further examined whether they were correlated with clinical manifestations. As shown in Fig. 4, LTF, MPO and SOD2 were significantly negatively correlated with neonatal weight and gestational age. Moreover, GSR was significantly negatively correlated with all three clinical manifestations.

### The four biomarker proteins were prognostic factors of gestational age

**Figure 5:**
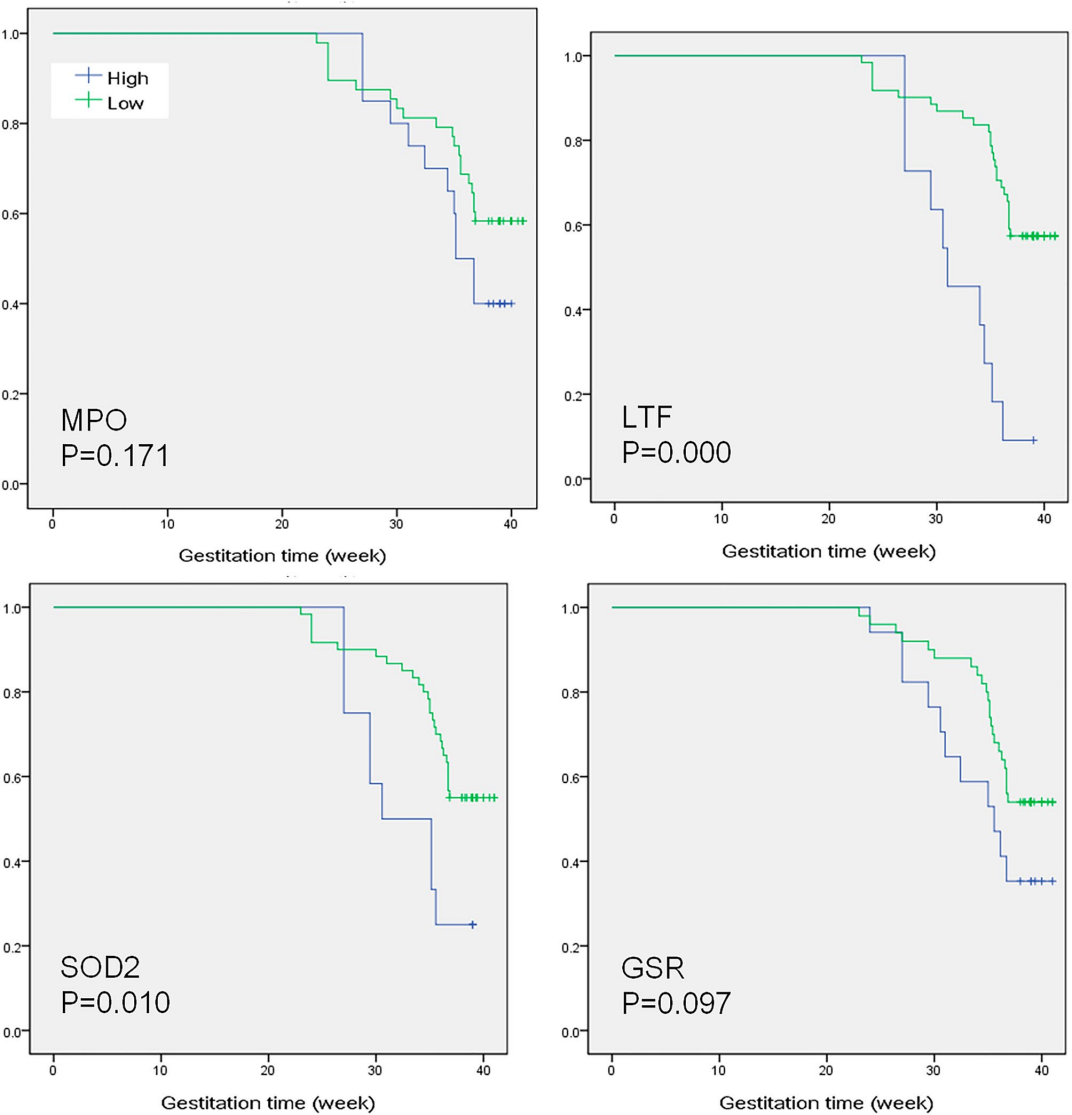
Survival rate analysis. For each participant, we inputted only the protein biomarker concentration and gestational age without providing FT or PT information. Then, we conducted survival rate analysis by mimicking the assays used in tumor biology. This analysis was performed with SPSS. The *p* values were calculated with the log-rank test.

Since the four biomarkers were either significantly correlated with gestational age or significantly abundant between the two sets, we further investigated whether the concentrations of the four biomarker proteins can be used to distinguish gestational age. In tumor biology, survival rate analysis is usually applied to investigate whether some elements contribute to the prognosis and regulation of survival time^23–25^. By mimicking the survival rate analysis, we investigated whether the concentrations of the four biomarkers contributed to gestational age without labeling the samples as FTB or PTB. As shown in Fig. 5, a higher abundance of LTF and SOD2 significantly resulted in shorter gestational age. In MPO and GSR, a similar phenomenon was observed although their *p* values were not yet significant. This result was consistent with Fig. 4.

### The PTB prediction model

**Figure 6:**
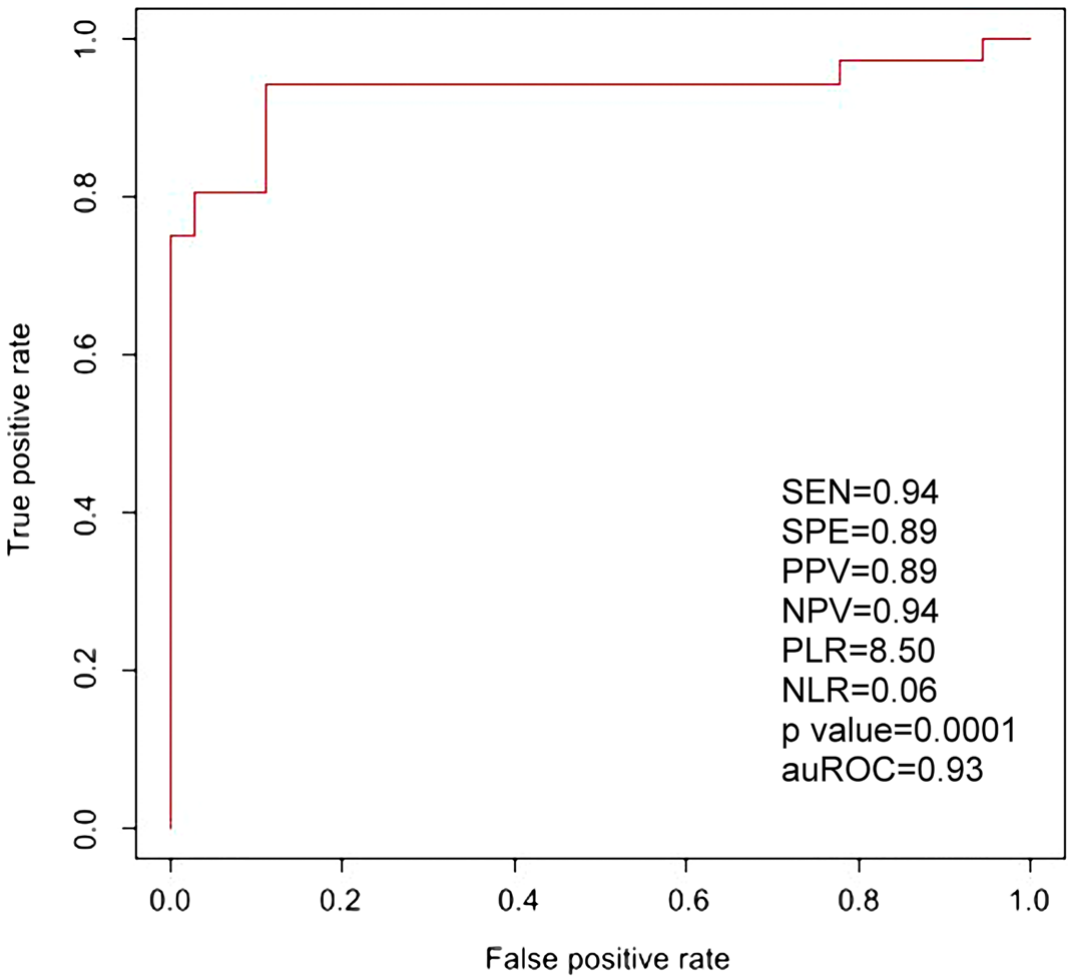
Prediction model of preterm birth based on the concentrations of four protein biomarkers. We used the concentrations of the four biomarker proteins to train the SVM algorithm, resulting in a high-performance prediction model with an auROC of 0.935 and a *p* value of 0.0001. Other parameters were also illustrated. SEN, SPE, PPV, NPV, PLR and NLR denote sensitivity, specificity, positive predictive value, negative predictive value, positive likelihood ratio and negative likelihood ratio, respectively.

Since the four biomarkers were differentially abundant between PTB and FTB AF samples and their higher levels also contributed to shorter gestational age, we wonder whether they could be applied to predict PTB. Therefore, we used the abundances of the 4 proteins from 36 PT and 36 FT samples to train the SVM model. It turned out that we obtained a prediction model of which the numbers of true positive, false negative, false positive and true negative were 34, 2, 4 and 32, respectively. As a result, the sensitivity, specificity, positive predictive value, negative predictive value, positive likelihood ratio and negative likelihood ratio were 0.94, 0.89, 0.89, 0.94, 8.50 and 0.06, respectively. In addition, as shown in Fig. 6, the prediction model has an auROC of 0.935 and a *p* value of 0.0001, reflecting a high-performance result. This prediction model allows us to predict PTB in advance by measuring the concentrations of the four biomarker proteins in amniotic fluid.

### The possible functions and interactions of the protein biomarkers

We also investigated the possible regulation mechanisms or interactions of the four biomarker proteins with Ingenuity Pathway Analysis (IPA, Qiagen). As a result, between LTF and MPO, there is a direct connection that LTF activates MPO (Supplementary Fig. 1). For GSR, its major function is to maintain a reductive environment by catalyzing glutathione disulfide into glutathione (Supplementary Fig. 2), which is consistent with previous studies^26,27^. For SOD2, no clear interaction with the other three proteins or no obvious pathway was identified.

## Discussion

Preterm birth (PTB) is the leading cause of neonatal morbidity and mortality if intensive medical care is not available for PTB neonates. Despite many possible pathogenic causes of PTB, early prediction of a PTB case enables medical personnel to make preparations in advance and to protect the neonate from the subsequent health risk. Therefore, invasive or noninvasive biomarkers for PTB are commonly investigated, including fetal fibronectin, placental alpha-microglobulin-1 and phosphorylated insulin-like growth factor binding protein-1^13,14^. Although the combination of these biomarkers enhanced the predictive efficacy^28^, the overall performance of these biomarkers are behind satisfactory. Therefore, we conducted this study by collecting AF protein samples the second trimester of gestation followed by combining global protein screening with mass spectrometry and specific validation with ELISA. It turned out that we identified four PTB biomarkers, including LTF, SOD2, GAR and MPO. We further used support vector machine algorithm and the concentrations of the four biomarkers to develope a high-performance prediction model with an auROC 0.935. At the second trimester of gestation, by collecting 10 ml of amniotic fluid and measuring the concentrations of the four biomarker proteins, the prediction model may accurately determine if a PTB case.

In our previous study, apolipoprotein A-IV, lumican and kininogen-1 in the amniotic fluid samples of preterm participants were different from those of full-term participants^18^. In addition, the placental protein 14 profile in amniotic fluid was suggested as a potential biomarker of premature rupture of the membrane^29^. However, these results were derived with traditional proteomics technology. Traditional proteomics technology requires 2-D gel, image scan and image analysis, costing much time and labor. Therefore, only a small fraction of proteins can be examined simultaneously. In this study, we applied isobaric tag for relative and absolute quantitation (iTRAQ) gel-free proteomics. This technology allowed us to identify and quantify proteins simultaneously in different samples, facilitating protein identification and quantification in amniotic fluid samples. Therefore, iTRAQ gel-free proteomics technology has been widely used in liquid biopsy-related studies^20,21^.

Based on the ELISA result, only LTF and SOD2 reached statistical significance. Although not yet reaching statistical significance, GSR and MPO had *p* value 0.0876 and 0.0650, respectively. Therefore, in addition to LTF and SOD2, GSR and MPO were also included in developing the PTB prediction model which had an auROC 0.935. Actually, we also developed a prediction with LTF and SOD2 only. As a result, we acquired a prediction model with auROC 0.7963. Such result is consistent with the concept that more reliable predictors usually leads to a better prediction model, with higher auROC.

In this study, we identified four protein biomarkers of PTB, including lactotransferrin (LTF), glutathione-disulfide reductase (GSR), myeloperoxidase (MPO) and superoxide dismutase 2 (SOD2). Briefly, these four protein biomarkers are associated with oxidative stress or infection. LTF is a member of the transferrin glycoprotein family and is widely distributed throughout biofluids, including milk, saliva, tears and so on^30^. Although it has multiple functions, the most well-known function of LTF is its antimicrobial activities^31^. Therefore, LTF was also an infection marker in amniotic fluid^32,33^. MPO is highly expressed in neutrophils and is involved in innate immunity by catalyzing the production of hypohalous acids with antimicrobial activity^34^. The plasma level of MPO is also an early risk predictor of myocardial infarction in patients with chest pain^35^. In the pregnant women with microbial invasion in the amniotic cavity, the AF level of MPO is also significantly elevated^36^.

SOD2, belonging to the iron/manganese superoxide dismutase gene family, is an indicator of apoptosis activity and oxidative stress^37^. Than et al.reported that SOD2 maintained higher mRNA levels in the chorioamniotic membranes in the pregnant women with acute chorioamnionitis or preterm labor^38^. GSR catalyzes the conversion of glutathione disulfide into glutathione so that living cells maintain a reductive environment^26,27^.

Although the final prediction model had a high performance, there are many weaknesses in this current study. First of all, the sample size is limited. Studies related to early prediction of PT birth needs to collect samples before sample category information, PT or FT birth, is available. Only when neonates are delivered, the collected samples can be classified into PT or FT birth, and the associated analyses can be done. In addition, in order to collect PT samples, almost 10 times more FT samples are unavoidably to be collected (Fig. 1), which costs much money and efforts. These limitations usually lead to a small sample size. Secondly, the predictive model was not examined for its performance in another independent cohort. Although we applied tenfold cross validation strategy in developing the prediction model, overfitting could still a problem. Only the validation with an independent cohort, overfitting problem can be excluded. And, the performance of prediction model can be confirmed.

## Conclusions

In this study, by global screening and specific validation, we identified four protein biomarkers of PTB in the amniotic fluid, including LTF, SOD2, GSR and MPO. Further analyses demonstrated that the four biomarkers were correlated with gestational age and neonatal weight, and LTF and SOD2 were also the prognostic indicators of gestational age. By using the abundance data, we developed a high-performance prediction model of PTB. This study demonstrated that the abundances of specific proteins in amniotic fluid facilitated the early prediction of PTB, enabling medical intervention to be applied in advance.

## Supplementary Information

Supplementary Information.

## Data Availability

Please contact Prof. Sung-Chou Li (raymond.pinus@gmail.com) for data requests.

## Abbreviations

PTB: Preterm birth
FTB: Full-term birth
ELISA: Enzyme-linked immunosorbent assay
LTF: Lactotransferrin
GSR: Glutathione-disulfide reductase
MPO: Myeloperoxidase
SOD2: Superoxide dismutase 2
SVM: Support vector machine
iTRAQ: Isobaric tag for relative and absolute quantitation
GO: Gene ontology
