# Author Correction: The abundances of LTF and SOD2 in amniotic fluid are potential biomarkers of gestational age and preterm birth

**DOI:** 10.1038/s41598-023-32640-2

**Published:** 2023-03-31

**Authors:** Te-Yao Hsu, Hsin-Hsin Cheng, Kuo-Chung Lan, Hsuan-Ning Hung, Yun-Ju Lai, Chih-Chang Tsai, Wen-Lang Fan, Sung-Chou Li

**Affiliations:** 1 Department of Obstetrics and Gynecology, Kaohsiung Chang Gung Memorial Hospital and, Chang Gung University College of Medicine, Kaohsiung, Taiwan; 2 Department of Obstetrics and Gynecology, Jen-Ai Hospital, Taizhong, Taiwan; 3 Department of Medical Research, Kaohsiung Chang Gung Memorial Hospital and Chang Gung University College of Medicine, 12th Floor, Children’s Hospital, No.123, Dapi Rd, Niaosong District, Kaohsiung, 83301 Taiwan; 4 Department of Medical Education and Research, Kaohsiung Veterans General Hospital, 4th Floor, No.386, Dazhong 1st Rd, Zuoying District, Kaohsiung, 813414 Taiwan

Correction to: *Scientific Reports* 10.1038/s41598-023-31486-y, published online 25 March 2023

The Funding section in the original version of this Article was incomplete.

"This study was supported by Chang Gung Memorial Hospital (CMRPG8A0501).”

now reads:

“This study was supported by Chang Gung Memorial Hospital (CMRPG8A0501 and CMRPG8L0101).”

The original Article has been corrected.

