# Supplementary material for: The abundances of LTF and SOD2 in amniotic fluid are potential biomarkers of gestational age and preterm birth
Source: Sci Rep. 2023 Mar 25;13:4903. doi: 10.1038/s41598-023-31486-y (PMC10039869; doi:10.1038/s41598-023-31486-y)

**Supplementary Figure 1. The interaction between LTH and MPO.** We used IPA to investigate possible regulation mechanisms or interactions of the four biomarker proteins. It turned out that LTF directly activated MPO so that this positive interaction (both LTF and MPO remained at higher levels in the AF samples of PA patients) was onserved between LTF and MPO.





**Supplementary Figure 2. The key regution function of GSR in maintaining an reductive environment.** GSR catalyzed the conversion from glutathione disulfide into glutathione, keeping glutathione complex at the the reductive state.


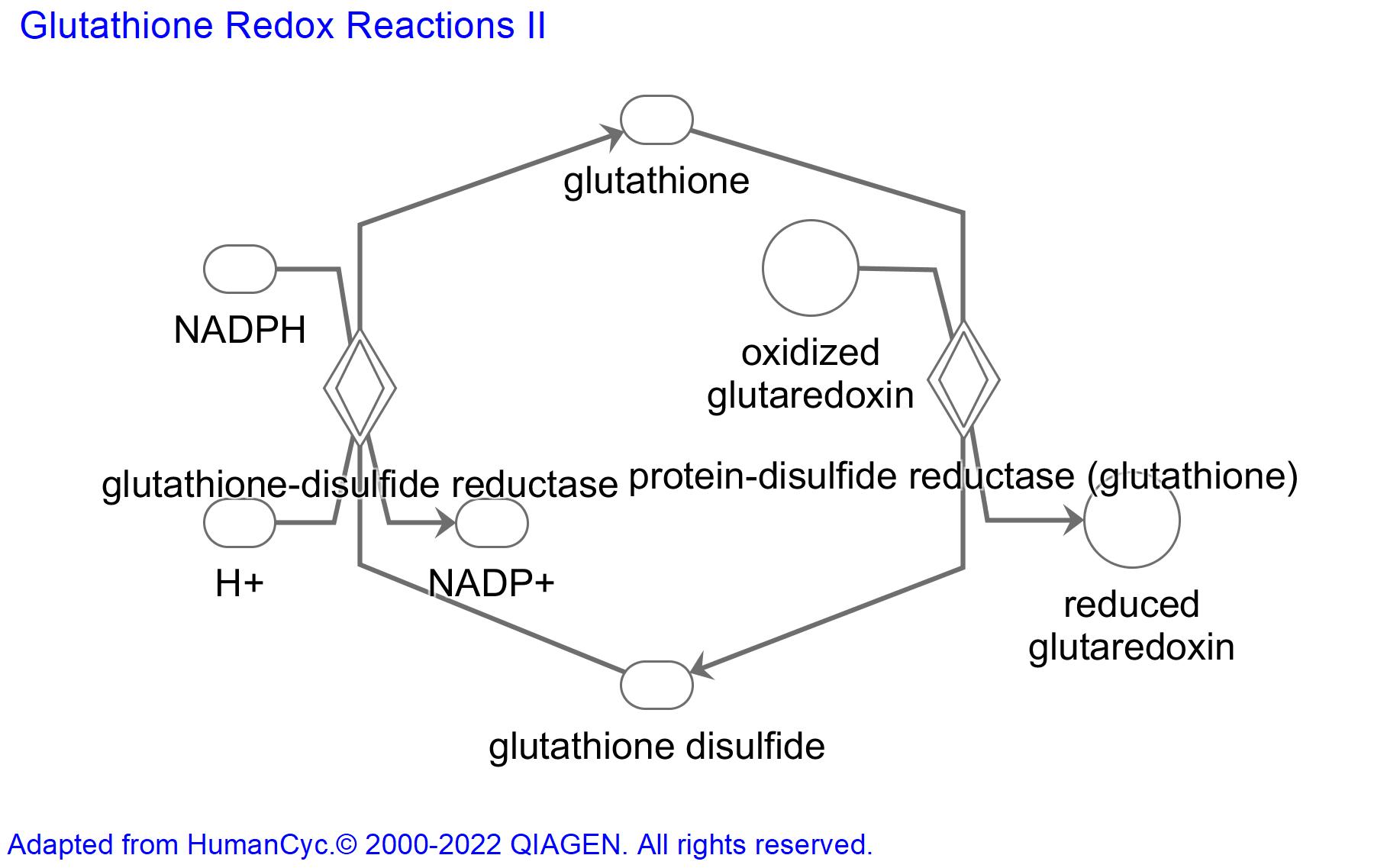

Supplement: Supplementary file 1 — Supplementary Information. [file 41598_2023_31486_MOESM1_ESM.doc]
